# Is BMP-7 a promising diagnostic and prognostic biomarker for pediatric obstructive uropathy?

**DOI:** 10.1186/s12894-026-02151-z

**Published:** 2026-05-21

**Authors:** Mehdi Shirazi, Seyed Hossein Hosseini, Maryam Salehi, Sania Shirazi

**Affiliations:** 1 Department of Urology, School of Medicine, Shiraz University of Medical Sciences, Shiraz, Iran; 2 Histomorphomettery and Stereology Research Center, Shiraz University of Medical Sciences, Shiraz, Iran

## Abstract

**Background:**

Obstructive uropathy remains a major contributor to pediatric chronic kidney disease. Reliable biomarkers that predict disease progression or recovery are still lacking. Bone morphogenetic protein-7 (BMP-7), known for its anti-fibrotic and renoprotective properties, has been proposed as a promising biomarker in renal injury. This study aimed to assess serum and urinary BMP-7 levels in pediatric obstructive uropathy and to evaluate its potential diagnostic and prognostic significance.

**Methods:**

This prospective observational study enrolled 200 pediatric patients with obstructive uropathy (including UPJO, UVJO, and PUV) and 200 healthy controls. Serum and urinary BMP-7 levels were measured before and three months after surgery using a human BMP-7 ELISA kit (Zellbio). Comparisons were made between groups and between pre- and postoperative measurements using appropriate statistical analyses.

**Results:**

Urinary BMP-7 levels were marginally higher and serum BMP-7 levels slightly lower in patients than in controls; however, these differences were not statistically significant (*P* > 0.05). Postoperative evaluations revealed a minor decrease in urinary BMP-7 and a mild increase in serum BMP-7, but again without statistical significance. No significant variations were found among obstruction subtypes (UPJO, UVJO, PUV).

**Conclusion:**

While BMP-7 plays a recognized role in renal repair in experimental models, its clinical diagnostic and prognostic utility in pediatric obstructive uropathy appears limited. Future research incorporating additional biomarkers and longer follow-up may further elucidate BMP-7’s potential in predicting renal recovery and fibrosis modulation.

## Introduction

Obstructive uropathy is the obstruction of urinary flow within the urinary tract due to a structural or functional disorder. Urine backs up into one or both kidneys, causing hydronephrosis or a dilation of the renal pelvis and calyces [1]. Antenatal hydronephrosis is the leading abnormality detected by prenatal ultrasonography [2], occurring in approximately 0.5–2% of fetuses [3, 4]. While this condition may be isolated and transient, it can also be caused by serious, permanent conditions like high-grade congenital vesicoureteral reflux (VUR), ureteropelvic junction obstruction (UPJO), obstructive megaureter and posterior urethral valve( PUV) [2]. It should be noted that the main cause of pediatric chronic kidney disease is congenital obstructive nephropathy [5].

The management of antenatal hydronephrosis is controversial because imaging studies and biochemical markers often fail to predict the risk of disease progression and the need for surgery [5]. A conservative approach with close follow-up has been recommended for most patients with obstructive uropathy including UPJO [2]. Although more than half the cases resolve during pregnancy or the first year of life, some cases will require surgery [3]. Factors influencing the decision-making process include the degree and laterality (unilateral or bilateral) of hydronephrosis, the change in renal function, and the presence of hydroureter or lower urinary tract obstruction. Intervention is typically reserved for patients who show renal function deterioration, hydronephrosis worsening, or complicated urinary tract infections [6]. Nonetheless, high-quality evidence-based recommendations are still lacking, making the management of antenatal hydronephrosis a controversial issue [6]. The critical concern is that progression to end-stage kidney disease (ESRD) is usually during adulthood [5], so finding factors that predict the disease course is essential.

Certain biomarkers have shown promise in assessing the presence and severity of conditions like UPJO, with transforming growth factor-1 (TGF-1), N-acetyl-beta-d-glucosaminidase (NAG), and neutrophil gelatinase-associated lipocalin (NGAL) being the most studied urinary biomarkers [4, 7]. Another candidate is bone morphogenetic protein 7 (BMP-7), a member of the TGF-β protein superfamily. This protein inhibits TGF-β-dependent biological functions and has been used to prevent obstruction-induced renal injury from developing [8]. BMP-7 has recently attracted interest due to its decreased levels during renal injury development and its possible role in determining the reversibility of obstruction-induced renal injuries, as demonstrated in animal studies [8].

While BMP7 and its engineered forms (BMP7 + sEVs, HSA-BMP7) exhibit strong anti-fibrotic, anti-inflammatory, and metabolic benefits across liver, kidney, rheumatic, and diabetic disease models (9, 10, 11, 12, 13), clinical studies are scarce. To our knowledge, no study has esamined the role of BMP7 specifically in the context of urinary obstruction in pediatric patients, leaving a significant gap in understanding the potential diagnostic and therapeutic applications of BMP7 for this condition. Hence, we aimed to investigate BMP-7 as a biomarker in pediatric obstructive uropathy, assessing its diagnostic value by comparing urine and serum levels between patients and controls, as well as before and after surgery, to determine its role in predicting disease progression and the need for intervention. In particular, we examined whether BMP-7 levels increase or decrease in response to urinary tract obstruction, thereby providing further insight into its diagnostic and prognostic utility.

## Patients and methods

This prospective observational study aimed to evaluate BMP7 levels in pediatric patients younger than 18 years of age diagnosed with obstructive uropathy, including ureteropelvic junction obstruction (UPJO), ureterovesical junction obstruction (UVJO), and posterior urethral valve (PUV). Surgical indications for UPJO were based on progressive hydronephrosis observed on serial imaging, differential renal function below 40%, symptomatic obstruction (e.g., flank pain or febrile urinary tract infections), or functional decline noted during follow-up. For UVJO, surgery was indicated in cases of worsening hydronephrosis, ureteral dilation with poor drainage, recurrent febrile UTIs, or declining renal function demonstrated on diuretic renography. In PUV patients, intervention was performed following confirmation of bladder outlet obstruction via voiding cystourethrography and endoscopic identification of the valve, typically during the neonatal period or early infancy.

The study received approval from the Institutional Ethics Committee to ensure adherence to ethical standards for research involving minors.

### Study design and population

A total of 200 pediatric patients with confirmed obstructive uropathy were recruited for the study. All UPJO cases included in this study were unilateral. Inclusion criteria included patients under 18 years of age requiring surgical intervention for obstructive uropathy. Exclusion criteria encompassed individuals with a history of chronic kidney disease, autoimmune disorders, or other systemic conditions that could influence BMP7 levels, such as systemic lupus erythematosus (SLE), inflammatory bowel diseases (e.g., Crohn’s disease, ulcerative colitis), diabetes mellitus (Type 1 and Type 2), thyroid dysfunction (hypothyroidism or hyperthyroidism), hyperparathyroidism, cancers, obesity, hypertension, chronic infections, or liver diseases (e.g., cirrhosis or hepatitis). Estimated glomerular filtration rate (eGFR) was calculated preoperatively using the age-adjusted Schwartz formula, and patients with eGFR < 60 ml/min/1.73 m² were excluded. Patients with active febrile urinary tract infection at the time of sampling were excluded. Urine cultures were reviewed to ensure absence of active infection. Additionally, complete blood count (CBC) was assessed at the time of sampling, and none of the participants demonstrated leukocytosis suggestive of active systemic inflammation. Patients with a history of prior urinary tract surgeries were also excluded. A control group of healthy pediatric patients with no history of renal or urinary tract diseases was included for comparison.

### Sample collection and processing

Blood and urine samples were collected from each patient preoperatively and three months postoperatively. Urine samples were collected as standardized first-morning midstream specimens prior to surgical intervention to minimize circadian variation and hydration-related dilution bias. All samples were processed immediately after collection under uniform laboratory conditions. Following centrifugation, specimens were aliquoted and stored at − 80 °C until analysis. Each sample underwent a single freeze–thaw cycle prior to ELISA measurement. Serum was isolated from blood samples through centrifugation, and urine samples were similarly processed for analysis. The normal range for serum and urine BMP7 is 14–480 ng/ml. BMP7 levels were quantified in both serum and urine using a commercially available enzyme-linked immunosorbent assay (ELISA) kit specifically designed for human BMP7, manufactured by Zellbio. The Zellbio kit demonstrated sensitivity and specificity within acceptable ranges for clinical research purposes.

### BMP7 measurement and analysis

BMP7 levels were measured in the collected samples and compared to those of the control group.In patients diagnosed with ureteropelvic junction obstruction (UPJO), open pyeloplasty was performed. The double-J stent was removed eight weeks postoperatively. A recurrence rate of 3% was recorded; these cases were excluded from the study and replaced with newly enrolled participants. For patients with ureterovesical junction obstruction (UVJO), cutaneous ureterostomy or endoscopic management was performed in infants under one year of age, whereas ureteral reimplantation was carried out in patients older than one year. The double-J stent was removed eight weeks after surgery. A recurrence rate of 5% was noted; these patients were excluded from the study and substituted with new cases to maintain the study cohort. The postoperative measurements were performed 3 months after Double J stent removal post surgical intervention, a time point chosen based on prior research suggesting significant changes in BMP7 levels during the late recovery phase. This timeline allowed for the assessment of BMP7 dynamics in response to the resolution of urinary obstruction.

### Ethical considerations

The study adhered to ethical guidelines, ensuring the protection and privacy of all participants. Written informed consent was obtained from the parents or legal guardians of the pediatric patients involved in the study.

## Results

This study included 200 children with urinary tract obstruction and 200 children without obstruction. Among the obstructed group, 137 patients were diagnosed with ureteropelvic junction obstruction (UPJO). All UPJO cases were unilateral in presentation with a mean age of 3.5 years, 42 patients had ureterovesical junction obstruction (UVJO) with a mean age of 2.8 years, and 21 patients were diagnosed with posterior urethral valves (PUV), presenting at a mean age of 2 days.

Table 1 presents the differences in average age and gender distribution between the patient and control groups. There was no significant difference in mean age (P = 0.13) or gender distribution (P = 0.08) between the patient and control groups. 

**Table 1:** Demographic characteristics of the control group and patients with urinary tract obstruction

**Variable**	**Control (n=200)**	**UPJO (n=137)**	**UVJO (n=42)**	**PUV (n=21)**	**P-value**
Age, mean ± SD (range)	3.80 ± 3.46 yrs(1 mo – 12 yrs)	3.5 ± 1.8 yrs(6 mo – 7 yrs)	2.8 ± 1.5 yrs(1 mo – 5 yrs)	2 ± 1.2 days(1 – 4 days)	0.13
Sex, Boys (%)	106 (53.0%)	60 (43.8%)	24 (57.1%)	21 (100%)	0.08†

*Abbreviations:* *UPJO *Ureteropelvic Junction Obstruction, *UVJO* Ureterovesical Junction Obstruction, *PUV *Posterior Urethral Valves, *mo *months, *SD *Standard Deviation

†Chi-square test for overall gender distribution across groups

Table 2 shows a comparison of urinary and serum BMP7 levels between patients with urinary tract obstruction and healthy controls before surgery. Although urinary BMP7 levels were slightly higher and serum levels slightly lower in patients compared to controls, these differences were not statistically significant (P = 0.37 for urine, P = 0.65 for serum). This suggests that BMP7 levels at baseline do not significantly distinguish obstructed patients from non-obstructed individuals. 

**Table 2:** Differences in average BMP7 levels in urine and serum between patient and control groups before surgery

**Variable**	**Patient Group (n=200)**	**Control Group (n=200)**	**P-value**
BMP7 (Urine)	177.07 ± 34.44 ng/ml	172.73 ± 30.25 ng/ml	0.37
BMP7 (Serum)	43.17 ± 27.76 ng/ml	44.78 ± 22.96 ng/ml	0.65

No statistically significant differences were observed between the groups (*P *> 0.05)

Table 3 presents the differences in average BMP7 levels in urine and serum between the control group and patient subgroups (UPJO, UVJO, and PUV) prior to surgery. Overall, the average BMP7 level in urine was higher in patients compared to controls, while serum BMP7 levels were lower in patients. Specifically, the UPJO subgroup exhibited the highest urinary BMP7 levels, followed by UVJO and PUV, whereas serum BMP7 levels were lowest in the PUV group. Despite these variations, none of the differences between patient subgroups and the control group reached statistical significance (P > 0.05).

**Table 3:** Differences in average BMP7 levels in urine and serum between patient subgroups and control group (before surgery)

**Group**	**Urine BMP7 (ng/ml) Mean ± SD**	**Serum BMP7 (ng/ml) Mean ± SD**	**P-value**
Control	172.73 ± 30.25	44.78 ± 22.9	–
UPJO (n=137)	180.12 ± 33.5	42.67 ± 25.1	>> 0.05
UVJO (n=42)	170.45 ± 36.2	45.03 ± 28.6	> 0.05
PUV (n=21)	165.90 ± 31.8	39.84 ± 29.3	> 0.05

Differences between the control group and all patient subgroups were not statistically significant (*P *> 0.05)

Table 4 displays the differences in average BMP7 levels in urine and serum within the patient group before and after surgery. The results indicate that the average BMP7 level in urine decreased post-surgery compared to pre-surgery, but this reduction was not statistically significant (P=0.19). Similarly, the average BMP7 level in serum increased post-surgery compared to pre-surgery, but this change was also not statistically significant (P=0.61). 

**Table 4:** Differences in average BMP7 levels in urine and serum within the patient group before and after surgery

**Variable**	**Before Surgery**	**After Surgery**	**P-value**
BMP7 (Urine)	177.07 ± 34.44 ng/ml	173.97 ± 33.88 ng/ml	0.19
BMP7 (Serum)	43.17 ± 27.76 ng/ml	43.64 ± 26.80 ng/ml	0.6

Changes in BMP7 levels before and after surgery were not statistically significant (*P *> 0.05)

Table 5 presents the changes in average BMP7 levels in urine and serum before and after surgery within each patient subgroup: UPJO, UVJO, and PUV. Across all subgroups, a slight decrease in urinary BMP7 levels and a slight increase in serum BMP7 levels were observed postoperatively. For instance, in the UPJO group, urinary BMP7 decreased from 180.12 ± 33.5 ng/ml to 175.90 ± 32.7 ng/ml, while serum BMP7 increased from 42.67 ± 25.1 ng/ml to 43.25 ± 24.6 ng/ml. Similar trends were seen in the UVJO and PUV groups. However, none of these changes were statistically significant (P > 0.05), indicating that surgical correction did not lead to meaningful short-term alterations in BMP7 levels within these subgroups.

**Table 5:** Differences in average BMP7 levels in urine and serum within patient subgroups before and after surgery

**Group**	**Urine BMP7 Before Surgery**	**Urine BMP7 After Surgery**	**Serum BMP7 Before Surgery**	**Serum BMP7 After Surgery**	**P-value**
UPJO (n=137)	180.12 ± 33.5	175.90 ± 32.7	42.67 ± 25.1	43.25 ± 24.6	> 0.05
UVJO (n=42)	170.45 ± 36.2	167.80 ± 35.5	45.03 ± 28.6	45.60 ± 27.9	> 0.05
PUV (n=21)	165.90 ± 31.8	160.75 ± 30.2	39.84 ± 29.3	40.55 ± 28.8	> 0.05

No statistically significant differences were observed before and after surgery within any subgroup (*P *> 0.05)

## Discussion

This study of 200 children with UTO and 200 controls found no significant differences in urinary or serum BMP7 levels between groups, across obstruction subtypes, or before and after surgery, although non-significant trends were observed with slightly higher urinary and lower serum BMP7 in obstructed patients and a postoperative shift toward lower urinary and higher serum levels. 

Obstructive uropathy is a condition in which there is a blockage or obstruction in the urinary tract that interferes with the normal flow of urine, which can dilate the renal pelvis and damage the kidney tissue, eventuating in renal failure if not treated [14]. In unilateral obstruction, where 20–40% of patients risk progressive renal decline, the key challenge for pediatric urologists is identifying which children need early surgery to preserve function versus those likely to recover spontaneously [15]. Chronic urinary obstruction is also known to cause fibrosis in the affected renal tissue, which can further contribute to the progressive decline in renal function by replacing functional parenchyma with scar tissue [16]. Fibrosis in obstructive uropathy results from renal ischemia and chronic obstruction, which lead to a reduction in blood flow and the activation of fibrogenic pathways. Key mechanisms include the activation of transforming growth factor-beta (TGF-β) and Smad3 signaling, promoting fibrosis and myofibroblast activation. Oxidative stress and inflammation further exacerbate this process, while epithelial-to-mesenchymal transition (EMT) contributes to renal damage [17].

Recent studies have highlighted the role of urinary biomarkers in noninvasively assessing renal injury caused by obstruction [18]. Studies investigating biomarkers like Neutrophil Gelatinase Associated Lipocalin (NGAL) and alpha-Glutathione S-transferase (GST) ratios in urine have revealed patterns consistent with renal impairment, providing valuable insights into obstruction-induced renal injury. This evidence underscores the potential of these biomarkers in evaluating the extent of renal damage caused by obstruction and in informing therapeutic decisions [7]. In the context of urinary biomarkers, BMP7 offers a promising complement to the extensively studied TGF-β pathway. BMP7 not only serves as a potential diagnostic marker but also plays a critical therapeutic role, particularly in kidney injuries associated with obstructive uropathies. Studies in murine models demonstrate that BMP7 levels decrease during the development of renal injuries and are upregulated during recovery, making it a valuable indicator of renal injury severity and recovery potential. Therapeutically, BMP7 has been shown to counteract the profibrotic effects of TGF-β by activating BMP-7-dependent Smad proteins (Smad1/5/8), which suppress the TGF-β/Smad2/3 signaling pathway. Exogenous BMP7 treatment accelerates renal repair, promotes resolution of fibrosis, and restores renal architecture even in cases of prolonged obstruction. These findings underline the dual role of BMP7 in both diagnosing and enhancing the treatment of renal injuries, highlighting its potential as a urinary biomarker and therapeutic target in clinical applications [8]. 

In light of these insights into BMP7’s role in renal injuries, further studies have continued to explore its therapeutic potential in other fibrotic and inflammatory conditions. Takano’s study on HSA-BMP7 in mouse models of liver fibrosis revealed its ability to reduce inflammation and fibrosis by inhibiting TGF-β signaling in both BDL and CCl4 models. Similarly, Smith's study underscored BMP7’s efficacy in kidney diseases such as diabetic nephropathy and lupus nephritis, where it reduced fibrosis and inflammation by counteracting TGF-β signaling and promoting tissue regeneration [10, 11]. In parallel, other research by Zhu and Narasimhulu highlighted BMP7’s therapeutic role in different models of fibrotic and metabolic diseases. Zhu’s study demonstrated that BMP7-loaded extracellular vesicles (BMP7+sEVs) reversed liver fibrosis by targeting hepatic stellate cells and inhibiting TGF-β/Smad signaling, while Narasimhulu’s work in diabetic mice showed BMP7’s ability to alleviate sarcopenia and improve muscle function by reducing inflammation and lipid accumulation [9, 12]. In the context of diabetic kidney disease (DKD), BMP-7 plays a crucial role by reducing fibrosis and inflammation. By upregulating SnoN, which prevents fibrosis and epithelial-mesenchymal transition (EMT) in kidney cells, BMP-7 activates the Smad1/5 pathway to maintain SnoN levels, offering a potential therapeutic strategy for preserving kidney function in diabetes [13]. 

BMP7-loaded small extracellular vesicles (BMP7+sEVs) have been shown to effectively target liver fibrosis, accumulating in fibrotic liver tissue and selectively targeting activated hepatic stellate cells (aHSCs). This treatment has demonstrated a reduction in fibrosis markers, improvement in liver function, and inhibition of aHSC migration and proliferation, suggesting that BMP7+sEVs offer a promising therapeutic approach for liver fibrosis [9]. In line with these findings, HSA-BMP7 has also exhibited strong anti-fibrotic effects in both acute and chronic liver fibrosis models. By reducing collagen deposition and fibrosis markers, along with having a longer half-life than BMP7, HSA-BMP7 suppresses hepatic stellate cell activation and TGF-β signaling. These findings underscore its potential as an effective therapy for liver fibrosis [10]. Moreover, BMP7’s anti-fibrotic capabilities extend beyond the liver. In models of kidney and rheumatic diseases, BMP7 has been shown to protect key cell types, such as podocytes, by modulating critical pathways like TGF-β, thereby preventing fibrosis progression. BMP7’s ability to improve renal function and tissue remodeling further highlights its promise as a therapeutic for various fibrotic conditions [11]. In addition, BMP-7’s therapeutic effects have been explored in diabetic mice, where it significantly improved metabolic and inflammatory conditions, including blood glucose levels, lipid profile, and muscle health. Furthermore, BMP-7 exhibited anti-inflammatory and anti-fibrotic effects, particularly in diabetes-induced muscle dysfunction, which could have broader implications for managing complications in diabetic patients [12]. In the context of diabetic kidney disease (DKD), BMP-7 plays a crucial role by reducing fibrosis and inflammation. By upregulating SnoN, which prevents fibrosis and epithelial-mesenchymal transition (EMT) in kidney cells, BMP-7 activates the Smad1/5 pathway to maintain SnoN levels, offering a potential therapeutic strategy for preserving kidney function in diabetes [13]. Together, these studies further underscore BMP7's role as an anti-fibrotic and anti-inflammatory agent.

While previous studies have emphasized the diagnostic and therapeutic potential of BMP7 in various models of kidney injury, the present study aimed to investigate its relevance in the context of UTO and the postoperative period. Despite BMP7’s recognized role in renal repair and fibrosis attenuation, our findings did not demonstrate statistically significant differences in BMP7 levels between UTO patients and healthy controls, nor between pre- and postoperative measurements within the patient group. This contrasts with the more pronounced shifts in BMP7 observed in other fibrotic conditions, suggesting that BMP7’s role in UTO may be more nuanced and dependent on factors such as the timing of intervention and the specific nature of the injury. It is also possible that BMP7-related changes occur primarily at the tissue level and may not be readily detectable in serum or urine, further underscoring the complexity of BMP7’s role in renal pathology and recovery. In addition, the cellular origin of urinary BMP-7 cannot be definitively determined based on compartmental measurements alone. BMP signaling is active in multiple segments of the urinary tract, including renal tubular epithelium and bladder urothelium. Experimental studies have demonstrated activation of profibrotic pathways such as TGF-β signaling in bladder outlet obstruction models, and given the known regulatory interplay between TGF-β and BMP pathways, it is biologically plausible that BMP signaling may also be involved at the bladder level [19]. However, in the absence of tissue-level expression analysis (e.g., immunohistochemistry or transcriptomic profiling), attribution of urinary BMP-7 to a specific anatomical source remains speculative. Importantly, BMP-7 levels did not significantly differ across obstruction subtypes in our cohort, including cases of posterior urethral valves, suggesting that bladder-level pathology was unlikely to be the dominant determinant of urinary BMP-7 concentrations in this dataset. Future studies incorporating tissue-specific assessment are warranted to clarify the relative contribution of renal and lower urinary tract compartments.

Shirazi et al. investigated the relationship between biochemical markers and renal recovery in patients with ureteropelvic junction obstruction (UPJO), highlighting the diagnostic potential of molecular indicators during the post-surgical period. The study reported significantly elevated urinary levels of TNF-α and caspase-3 in patients prior to surgical intervention compared to healthy controls, which returned to near-normal levels six months postoperatively. These molecular changes were accompanied by measurable improvements in renal morphology and function, including reductions in anteroposterior (AP) diameter and increases in cortical thickness, suggesting a strong association between structural recovery and biomarker normalization. Although BMP7 was not assessed in that study, the results underscore the broader utility of biomarkers such as TNF-α and caspase-3 in monitoring renal healing and guiding postoperative follow-up [20].

Manson et al. examined the role of BMP-7 in a murine model of obstruction-induced renal injury, providing important evidence for its involvement in kidney repair mechanisms. The study demonstrated that BMP-7 expression was significantly elevated during the early phases of recovery following timely relief of obstruction, correlating with notable improvements in both renal structure and function. In contrast, delayed intervention leading to fibrotic progression was associated with diminished BMP-7 activity and impaired regenerative capacity. The activation of the BMP-7 signaling pathway was shown to be critical for reversing fibrosis, primarily through the inhibition of profibrotic mechanisms such as TGF-β-dependent signaling. Furthermore, administration of exogenous BMP-7 enhanced recovery outcomes in cases of advanced damage, reinforcing its therapeutic potential in renal injury [8].

Although Wang et al. reported that reduced renal function in UPJO patients was significantly associated with elevated MMP-7 and NGAL levels and decreased TIMP-2 levels, their study focused on markers of injury and fibrosis. In contrast, our study examined BMP7, a molecule primarily involved in renal repair and regeneration. Therefore, the findings are not directly comparable due to fundamental differences in biomarker selection and the biological pathways they represent. The absence of significant changes in BMP7 levels in our study may reflect its distinct role, which might not manifest during early or compensated stages of obstruction [21].

Patel et al. demonstrated that overexpression of MMP-7 in congenital renal dysplasia disrupts nephrogenesis by suppressing BMP7 signaling. In contrast, our study found no significant changes in BMP7 levels before and after surgical relief of obstruction, suggesting that BMP7 may not be significantly impaired in postnatal obstructive uropathy. These differences highlight the distinct pathophysiological contexts—congenital dysplasia versus acquired obstruction—and suggest that BMP7 dysregulation may play a more prominent role in developmental anomalies than in compensatory postnatal repair [22].

Unlike the patterns reported in animal models, our study did not detect significant postoperative changes in BMP-7 levels among human patients, suggesting that BMP-7 may not consistently reflect the resolution of obstruction in this clinical context. This could be attributed to various factors, including species differences in BMP-7 activity, timing, and methodological variations, such as biomarker measurement intervals. Moreover, our findings emphasize the stability of BMP-7 across pre- and post-surgical time points in UTO patients, suggesting that BMP-7 may not play as prominent a role in immediate post-obstruction recovery in human subjects or that the timeline for BMP-7’s involvement is delayed beyond the period assessed.

In line with this, Carine Smith's study emphasizes the critical role of the TGF-β/BMP-7 ratio in kidney disease outcomes, specifically lupus nephritis, where an elevated ratio was linked to poorer prognosis. Investigating the TGF-β/BMP-7 ratio in future studies could offer valuable insights into the interplay between these factors and their implications for kidney fibrosis, providing a clearer understanding of their relative contributions and paving the way for targeted therapeutic approaches [11].

Given the importance of accessible and noninvasive diagnostic tools, the economic feasibility of biomarker-based testing becomes a key consideration in clinical practice. A hypothetical cost-effectiveness model suggests that, in their current form, individual urinary biomarkers may be less effective and more costly than diuretic renography (DR) for predicting UPJO. However, as research progresses, a future biomarker panel could improve both diagnostic accuracy and cost-effectiveness by reducing the need for invasive renography, thus limiting radiation exposure [18].

To maintain focus on economically viable clinical advancements, Smith, du Toit, and Ollewagen highlight the significant financial barriers to BMP-7 research and drug development. They propose solutions such as advanced drug delivery systems, improved synthesis methods, and the integration of artificial intelligence to reduce clinical trial expenses. Furthermore, they advocate for personalized treatments based on patient genotyping to enhance both therapeutic outcomes and cost-efficiency [11].

One of the key limitations of our study was the inability to assess TGF-β levels alongside BMP-7 due to the unavailability of TGF-β detection kits. This restricted our ability to directly compare the interplay between BMP-7 and TGF-β in the context of obstruction-induced renal injury and recovery. Both BMP-7 and TGF-β are critically involved in kidney repair and fibrosis, with BMP-7 being a potential counter-regulatory factor to the profibrotic actions of TGF-β. Given that several studies suggest a functional interaction between BMP-7 and TGF-β signaling pathways in the progression and modulation of renal fibrosis, the inability to assess both markers simultaneously may have limited our capacity to clarify their respective contributions to the underlying pathophysiological mechanisms of urinary tract obstruction and the effects of its surgical resolution. Future studies incorporating both BMP-7 and TGF-β levels in conjunction with other biomarkers may provide a more comprehensive understanding of the molecular dynamics at play and help clarify the potential for targeted therapies that modulate these pathways. Additionally, because only serum and urinary BMP-7 levels were measured, the study could not determine the precise anatomical or cellular source of urinary BMP-7 (renal tubular versus lower urinary tract origin), which may limit the mechanistic interpretation of the findings. While first-morning urine samples were used to reduce hydration-related variability, creatinine-adjusted normalization was not performed. Standardized sampling helps limit dilutional bias; however, the absence of normalization may represent a methodological limitation and should be considered when interpreting urinary BMP-7 levels. Patients with established CKD were excluded; nonetheless, minor variations in renal function within the non-CKD range were not controlled for using multivariate adjustment and may have influenced BMP-7 concentrations. Clinically active infections were excluded and no leukocytosis was observed; however, subtle localized urinary tract inflammation cannot be entirely ruled out and may have influenced biomarker levels. 

## Conclusion

While the BMP-7 pathway may play a significant role in renal recovery in certain experimental models, our study indicates that BMP-7 levels alone might not serve as a reliable clinical marker of recovery post-UTO surgery in humans. Future research could benefit from investigating BMP-7 alongside other markers or over extended timeframes to better understand its relevance and timing in renal recovery, especially as new insights into therapeutic BMP-7 activation are considered for clinical applications.

## Data Availability

The datasets generated and analyzed during the current study are available from the corresponding author upon reasonable request.

## Abbreviations

BMP-7: Bone morphogenetic protein 7
UPJO: Ureteropelvic junction obstruction
UVJO: Ureterovesical junction obstruction
PUV: Posterior urethral valves
UTI: Urinary tract infection
ESRD: End-stage renal disease
ELISA: Enzyme-linked immunosorbent assay
SD: Standard deviation
